# Economic Feasibility of Implementing Stunning for Farmed Fish in the EU: A Multi-Species Assessment

**DOI:** 10.3390/ani15192812

**Published:** 2025-09-26

**Authors:** Griffin Carpenter, Myriam Vanderzwalmen, Helen Lambert

**Affiliations:** 1 Independent Researcher, 1050 Brussels, Belgium; griffincarpenter@gmail.com; 2 Eurogroup for Animals, 1000 Brussels, Belgium; 3 Animal Welfare Consultancy, Kingsteignton, Devon TQ12 3BW, UK

**Keywords:** aquaculture, economic feasibility, humane slaughter, electrical stunning, in-water stunning, dry-stunning, common carp, rainbow trout, European seabass, gilthead seabream

## Abstract

Stunning of farmed fish prior to slaughter is increasingly recognized as a key animal welfare priority, yet uptake remains limited in the EU aquaculture sector. While the effects of different stunning methods on fish welfare are the subject of significant recent research, the effect on aquaculture businesses remains unclear. Therefore, this study assesses the economic feasibility of implementing electrical stunning for four species where it is not currently routine: carp, trout, seabass, and seabream. Using a granular cost model across 17 country–species–system combinations, and cost data from 2018 to 2020, the impact of introducing in-water and dry electrical stunning systems under various cost pass-through and sensitivity scenarios is evaluated. Results show that while stunning increases the production costs, under realistic assumptions, 16 out of 17 segments remain profitable, with the one unprofitable segment already being unprofitable under business-as-usual conditions. Three trout systems even experience cost savings due to reduced labor requirements. Sensitivity analyses confirm the robustness of these findings across plausible increases in operating costs and financing assumptions. Even under a 0% cost pass-through, 16 segments still remain profitable. These results provide timely, policy-relevant evidence to support species-specific welfare legislation, while identifying segments that may require targeted support for compliance.

## Simple Summary

Millions of farmed fish are killed every year in the EU without being stunned, despite science showing that they can feel pain and suffer. This study looked at whether it would be financially possible for fish farms to introduce stunning methods, such as electrical stunning, to improve fish welfare. Data from 17 different types of fish farming systems across Europe, focusing on carp, trout, seabass, and seabream, were reviewed. The results showed that although stunning adds some extra cost, most farms would still remain profitable, and in some cases, especially for trout, stunning could even save money by reducing labor requirements. Only carp farms appeared to be at higher financial risk, and one type of carp farm was already losing money before any changes. This suggests that introducing stunning is affordable for most fish farms, but some farms may need extra support. These findings can help EU policymakers create fair and effective laws that improve animal welfare without harming the fish farming sector.

## 1. Introduction

Stunning is widely recognized as a fundamental requirement for the slaughter of vertebrate animals, both legally and in terms of public expectation [1]. It ensures that animals are rendered unconscious before death, preventing unnecessary pain and suffering. While stunning is standard practice for terrestrial vertebrates, fish remain largely overlooked, as there is no regulatory requirement for stunning in the EU. Consequently, its application is uncommon in aquaculture and wild-capture fisheries, and fish are handled and killed whilst conscious, resulting in considerable suffering [2,3]. Furthermore, the slaughter methods commonly employed for fish, such as asphyxiation in air, ice, ice slurry, or CO_2_, with or without live chilling, are typically slow and not instantaneous, resulting in prolonged suffering for the fish [2,4,5].

Asphyxiation, as a slaughter method, involves the fish being pumped or netted into ice, ice slurry, or air, where they are left until they die, or in some systems until they are slaughtered by evisceration or exsanguination [2,6]. The experience of asphyxiating is highly aversive for fish, and they typically show vigorous escape behaviors [7,8,9] and elevated stress parameters [5,10]. Consequently, it is widely considered to be an inhumane killing method [7,10,11].

Carbon dioxide (CO_2_) narcosis may be used prior to exsanguination. This method involves the fish being transferred to a tank that is supersaturated with CO_2_, and sometimes also at a lower temperature [6,12]. The fish are typically left for several minutes before being removed and bled out by gill cutting. The process has been known to take up to 10 min for the fish to lose consciousness, although there are also reports of it taking up to 20 min for seabass [9,13]. As with asphyxiation, the fish display vigorous escape attempts before becoming immobilized [12,14,15,16,17]. However, they can remain conscious for some time following immobilization, which can mean they are processed and bled out whilst still conscious [17,18].

Some farmed species may also be slaughtered by exsanguination or decapitation, without stunning, meaning they are fully conscious when killed [11]. For example, flat species such as turbot have their gill arches cut and are left to bleed out, often in an ice slurry [19]. The European Food Safety Authority (EFSA) has stated that this method involves a prolonged period of consciousness, during which the fish show clear stress responses and escape behavior [11].

In the EU, fish, primarily carp, may also be sold live by supermarkets or other retailers to members of the public who may keep the fish alive for a period before slaughtering at home by asphyxiation, blows to the head, or exsanguination [4,20]. It is highly probable that the fish are subject to substandard housing, potentially for days, and then killed by an untrained person without any form of stunning [20].

These commonly used inhumane slaughter methods represent a significant welfare concern, particularly when the number of fish being killed is considered. Despite being sentient beings, fish numbers are still reported in terms of weight rather than individual numbers. Therefore, in this study, data from FishStatJ and Mood et al.’s midpoint estimates for weight [21,22,23] have been used to calculate estimated fish numbers based on tonnage, here and throughout, unless otherwise stated. In the EU, over 1 million tonnes of fish are harvested from aquaculture per year [21], which is estimated to equate to around 640 million fish annually. Aquaculture production is also growing, and according to the FAO, the growth rate of aquaculture production already exceeds that of most other food production systems and has the potential for further expansion [24]. Consequently, hundreds of millions of fish are subjected to an inhumane death every year in the EU alone.

**Figure 1:**
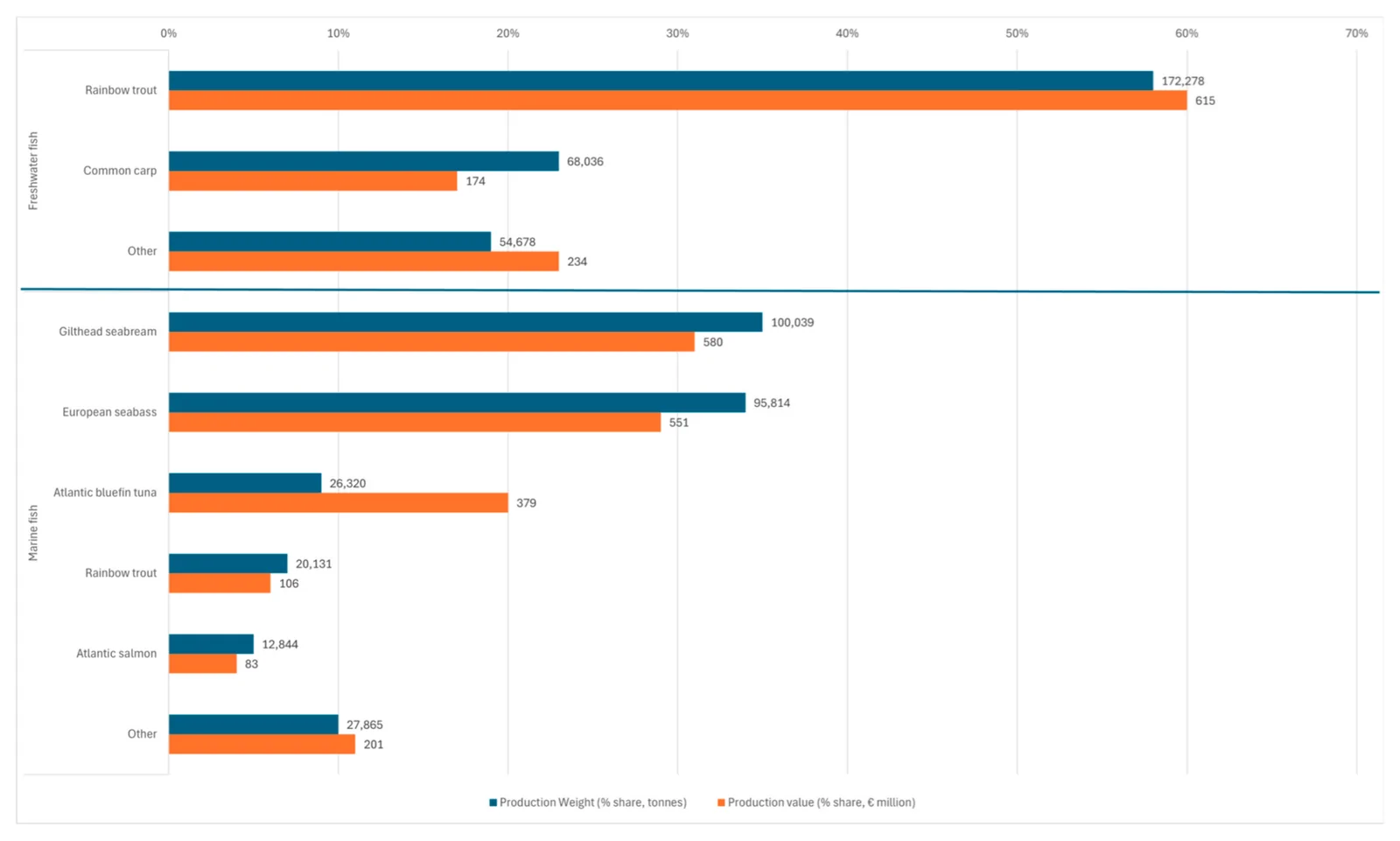
EU fish aquaculture production by species, showing the percentage contribution to total economic value (in EUR, orange bars) and total production weight (in tonnes, blue bars) (2021). Species are grouped by category for clarity, with a dividing line, and data labels show value (EUR) and weight (t).

Of the 200+ species in EU aquaculture, most of the production (weight and value) comes from twelve species, five of which are shellfish [21]. Another five are marine fish, and these five species constitute 90% of the marine fish production weight and 89% of the production value [25] (see Figure 1). Whereas for freshwater fish, the top two species constitute 81% of the production weight and 77% of the production value of freshwater fish production [25] (see Figure 1).

In 2020, the European Commission announced that it would be revising the EU’s animal welfare legislation to improve animal welfare standards in line with the latest scientific evidence, widen its scope, and make enforcement easier [26,27], specifically referencing the legislation on animal transport and slaughter [26]. Then, in January 2023, the Commission’s Inception Impact Assessment [28] suggested species-specific provisions for the killing of the five main species of farmed fish (*Atlantic salmon* (*Salmo salar*), common carp (*Cyprinus carpio*), rainbow trout (*Oncorhynchus mykiss*), European sea bass (*Dicentrarchus labrax*), and gilthead sea bream (*Sparus aurata*)) to better ensure the welfare of the species and to address existing gaps in legislation.

**Figure 2:**
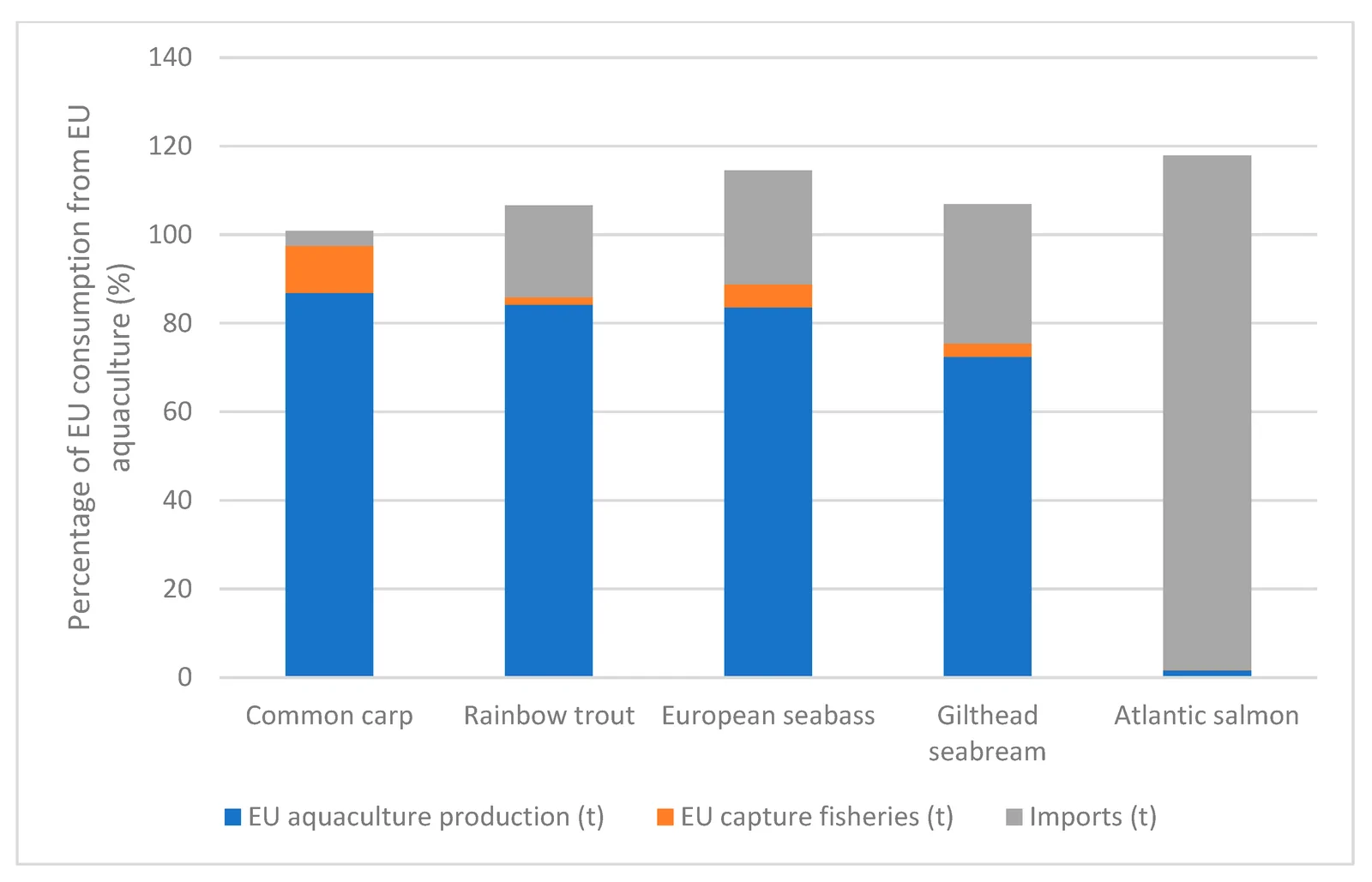
The proportion of apparent consumption supplied by EU aquaculture production, EU capture fisheries, and imports for the top five species. Each bar represents one species, with components stacked to show their relative contributions. Exports are not included in the stacked bars as they reduce available supply, and so for some species, production exceeds consumption (exceeding 100%). The export values are as follows: Common carp, 731 t of EU production; Rainbow trout, 15,626 t of EU production; European seabass, 12,035 t of EU production; Gilthead seabream, 8894 t of EU production; and Atlantic salmon, 195,167 t of EU production.

The impact of such measures would be considerable, as together, these five species represent 450,000 tonnes of annual production and EUR 2 billion in sales, which corresponds to 78% of the weight, 69% of the value, and 97% of the fish in EU fish aquaculture production [21,22,23,29]. For example, the EU produces 66 million trout, 286 million seabream, 213 million seabass, 45 million carp, and 2 million salmon each year [21,22,23,29]. Most of these fish are consumed within the EU, as comparatively little is exported [30] (Figure 2).

Whilst salmon are typically stunned using electrical or percussive stunning, the remaining four main species of farmed fish in the EU are routinely exposed to an inhumane death without stunning [2]. Percussive stunning is generally considered impractical for smaller-sized species and large numbers, making it widely unsuitable for carp, trout, sea bream, and sea bass [5,31]. However, electrical stunning can be used for a large number of fish, including those varying in size, making it more practical on a commercial scale [5]. Electrical stunning can take two forms, depending on whether the fish remains in the water or not. Wet or in-water stunning is particularly beneficial as the fish do not need to be removed from the water [5]. In contrast, in dry or in-air electrical stunning, the fish are first dewatered before being passed along an automatic conveyor belt, where they are exposed to an electrical current that renders them unconscious [12].

Electrical stunning could improve welfare by reducing stress and suffering at slaughter, when compared to the current practice of no stunning and prolonged death from asphyxiation [32]. However, electrical stunning has shown variable impacts in commercial and research settings, as some fish may not be immediately or consistently rendered unconscious [33,34,35,36,37], and so welfare outcomes can vary depending on species, size, system design, and operator skill [7,32,37,38]. Despite these limitations, electrical stunning could introduce a practical and meaningful improvement over no stunning. Practical steps are needed to move towards more humane and reliable slaughter practices, while further refinement and alternative methods can continue to be developed to achieve optimal welfare outcomes.

Despite the clear need for introducing stunning procedures, uptake in EU aquaculture remains limited, in part due to concerns over costs and operational feasibility. According to one study, producers of sea bream and sea bass consistently cited investment costs as being a “very important” barrier to implementing stunning mechanisms [39]. This finding is likely to be widespread across European aquaculture [39]. With that in mind, it is important to understand the economic implications of introducing stunning equipment and how investment costs can be distributed fairly to aid with uptake.

In 2017, the European Commission published its study into the animal welfare practices in European aquaculture for the transport and slaughter of farmed fish during 2009–2013 [2]. The study, hereafter referred to as the EC study, aimed to analyze the extent to which welfare issues were unresolved and considered the costs of adhering to good welfare practices, the economic situation, and the impact such improvements would have on competitiveness and other factors, such as product quality [2]. Using cost estimates for stunning equipment and data from the EU’s Data Collection Framework, it concluded that welfare improvements would have minimal impact on production costs [2]. More recently, a study by Essere Animali and Animal Ask, hereafter referred to as the EA/AA study, followed the same framework, estimating the effects of mandatory stunning on retail prices in Italy and Greece [40,41]. They found that the additional costs to consumers would be small, even with full-cost pass-through [40,41]. Therefore, both the EC and EA/AA studies highlighted that the costs of implementing pre-slaughter stunning represent only a small share of production costs and/or the final retail price.

Despite these findings, stunning mechanisms are still not commonly used in European aquaculture, and cost is still perceived as a significant barrier by some [39]. Therefore, in view of the political and ethical impetus to introduce mandatory stunning for farmed fish, the objective of this study is to assess the economic feasibility of implementing stunning mechanisms for the four main species in EU fish aquaculture, which are currently not routinely stunned prior to slaughter. This study expands on previous research by incorporating updated equipment costs, more recent economic data, and a refined analysis of cost distribution across the supply chain to assess the economic feasibility of mandatory stunning in the EU’s key aquaculture sectors.

## 2. Materials and Methods

### 2.1. Study Approach

This study uses the same framework as has previously been used to determine the economic feasibility of introducing stunning, but with significant changes that build upon these methods (Table 1). As with previous research in this area [28,40,41], this study uses a partial budget analysis to analyze the economic change before and after pre-slaughter stunning is implemented. A partial budget analysis is suited to this area, as it focuses specifically on the costs and benefits that change as a result of introducing pre-slaughter stunning in aquaculture [42,43]. Furthermore, the targeted approach allows for a clear assessment of the economic feasibility without requiring a full enterprise analysis, making it highly suited to this specific application. All calculations were carried out using Excel spreadsheets.

### 2.2. Study Scope

The scope of the current study is limited to the direct economic impacts on the EU’s fish aquaculture businesses. Indirect economic effects on the aquaculture supply chain, such as impacts on market access, consumer trends, and equipment manufacturing, are not covered in this analysis. Nor are the non-economic impacts, such as societal effects, or the welfare impact of introducing stunning to the fish being farmed.

The objective of this study is not only to estimate the costs of adding pre-slaughter stunning mechanisms to aquaculture systems but also to assess the economic feasibility of doing so. Therefore, the study also seeks to assess the impact of passing these additional costs on, and the extent to which they can be absorbed by the producers while still maintaining profitability (Table 2).

### 2.3. Species Scope

Atlantic salmon, common carp, rainbow trout, gilthead seabream, and European seabass are among the top species farmed in the EU in terms of production weight and economic value, and EU production of these species represents a considerable portion of the EU market (70% for seabass and seabream, 80% for trout, and 86% for carp, although only 1% for salmon since Brexit) [21]. These species are also considered in the European Commission’s Inception Impact Assessment as requiring the development of species-specific provisions for killing methods [28]. Of these top species, Atlantic salmon are the only species that are already routinely stunned in farms [2,44,45], and so were excluded from this study. This selection aligns with the European Commission’s focus and ensures the study addresses species where stunning adoption is not yet standard. For comparison, the EC study considered all five of these species, and the EA/AA study only considered sea bass, sea bream, and trout.

### 2.4. Segment Selection Method

The economic data for this study were captured from the biennial Scientific, Technical and Economic Committee for Fisheries (STECF)–Economic Report on the EU Aquaculture [46]. The 2022 report (published in 2023) covers the period of 2008 to 2020 and reports data by national totals and segments divided by species. The dataset is both comprehensive and standardized, thereby allowing for an accurate assessment regarding the economic feasibility of introducing pre-slaughter stunning to be made. However, due to gaps in data for Poland and Spain, data from the 2020 report were used for Spain [47], and an alternative economic data survey was used for Poland [48,49].

Relevant aquaculture segments (combination of country–species–production system) were selected using a threshold of >EUR 20 million in annual production value, based on the average of three years of data. This threshold was chosen as anything below this would likely be characterized by marginal operations, where aquaculture is a secondary income. For example, in Germany, carp farms are typically hobbies or second businesses rather than primary enterprises [48]. In total, 17 country–species–production system combinations were above this threshold and were included in the current study (Table 3). Collectively, they produce around 329,000 tonnes of fish annually, valued at EUR 1.5 bn and constituting around 522 million fish [21,22,23,29]. This accounts for around 57% of the total EU farmed fish production by weight, 52% of its value, and 81% of the EU’s farmed fish population by number [21,22,23,29].

The STECF economic data for aquaculture systems are reported as a total for each segment rather than for each enterprise [46]. Therefore, to calculate the performance of the average enterprise, the total value for the segment was divided by the number of enterprises. While this is standard methodology and is used in the EC study, it may mean that it is not fully representative of any enterprises that are either much larger or much smaller than the average. For Poland, where STECF data are not available, the economic data come from a study of the modal aquaculture enterprise (the most common), as opposed to the mean average enterprise [48].

### 2.5. Cost Estimation

From an economic perspective, the sticker price of capital equipment is not the most relevant consideration when calculating costs. If the equipment can be sold at the same price later (i.e., no depreciation), its net cost would be zero. Therefore, to determine the actual cost, the sticker price is necessary for calculating the loan costs (assuming the equipment is financed), along with factors such as depreciation, repair and maintenance costs, and any associated costs or savings from labor or energy.

Both the EA/AA and the EC studies included equipment costs in their analyses based on surveys and consultations with equipment manufacturers. More recent figures were obtained from one manufacturer (Optimar, Valderøya, Norway, personal communication, 27 July 2023) for an electro-stunner for post-dewatering, applicable for all species. Costs for four models were provided, and the average (mean) was used in the analysis. For all other stunning equipment, the most recent costs were included from the EA/AA and EC study (see Table 4). The equipment costs used in this study include the purchase price used for financing (assuming equipment is financed at a 5% interest rate over a 10-year loan period, as used in the EC study), the depreciation and maintenance rates, and the impact (hours per tonne) on labor costs (including start-up and training costs) (Table 4). Changes to energy costs were assumed to be insignificant (see Table 5 for rationale).

### 2.6. Assumptions

Assumptions regarding cost pass-through were based on trade data and market structure. It is assumed that market power, which is a company’s ability to influence the price of a product in a market, is half (50%) determined by market share and half (50%) determined by other factors. Therefore, for each species in the study, price transmission is assumed to be 0.5* EU production share of the EU market: 35% for seabass and seabream, 40% for trout, and 43% for carp. Several other methodological assumptions were included (see Table 5), which have the overall effect of overestimating the costs of implementing pre-slaughter stunning at slaughter, thereby reducing the projected economic feasibility of such measures. These methodological assumptions are a necessary component of analysis and are similar to those used in previous studies [2]. Where appropriate, these assumptions were also tested with sensitivity analyses (see Section 2.7).

### 2.7. Sensitivity Analyses

The methodological assumption that cost pass-through is 50% determined by market share and 50% by other factors was tested using sensitivity analysis to assess whether the economic feasibility was affected by different cost pass-through scenarios. Therefore, in addition to the market-based cost pass-through scenarios (35% for seabass and seabream, 40% for trout, and 43% for carp), low (0%) and high (100%) pass-throughs were also tested. The low pass-through scenario (0%) represented a situation where producers absorb all additional costs without increasing product prices, and a high pass-through scenario (100%) would be where all additional costs are fully reflected in the product price.

Sensitivity analyses were also performed to test the impact of increased operational costs associated with implementing stunning, such as increased energy usage or increased maintenance costs. For these, the total additional annual costs were increased by 10% and 20% relative to the base stunning scenario. These adjustments were applied across all segments under the market-based cost pass-through assumption before the effects on profitability were assessed.

### 2.8. Cost Analysis Metrics

The additional cost of stunning in aquaculture can be expressed across several metrics: (1) the total additional costs, (2) costs per unit of production, (3) as a percentage of current production costs, and (4) as a share of current price.

The additional costs of introducing stunning methods (1) are estimated by calculating the loan costs, depreciation, repair and maintenance costs, and any associated labor costs (see Section 2.5. ‘Cost estimation’). These costs are assumed to be incremental to existing operational expenditures and are, therefore, added to the farm’s overall cost structure.

To contextualize these additional costs and provide a more granular view, they are then expressed in terms of the impact on the cost per unit of production (cost per kg of fish) (2). For this metric, the total annual cost of implementation was divided by the baseline production value per enterprise (kg live weight) [46] for each segment (which had been updated to reflect 2024 rates of inflation [50]) and expressed as a per-unit cost (EUR/kg) for each segment. This metric provides a greater understanding of how the introduction of stunning methods will affect unit economics and whether the added costs are manageable in the context of production output.

Next, the additional costs are expressed as a percentage of current production costs (3) to assess the relative importance of stunning-related costs within the existing cost structure. This metric was calculated by dividing the baseline annual costs per enterprise (EUR) [46] for each segment (updated to reflect inflation [50]) and presenting them as a percentage of current production costs. This metric provides insight into how the cost increase compares to the farm’s existing expenses and whether it poses a significant challenge to profitability.

Finally, the additional costs are evaluated in terms of their impact on the price of the final product (4) to gauge whether the additional cost of stunning will likely be passed on to consumers or absorbed within the supply chain. To calculate the costs as a share of the final product price, the costs per unit of production were divided by the price per unit (BAU) according to the STECF dataset [46] (updated to account for inflation [50]). This metric aids in assessing the economic feasibility of stunning in terms of consumer prices and market competitiveness.

### 2.9. Economic Feasibility Assessment

The feasibility of implementing stunning mechanisms was assessed for each of the 17 country–species–system segments by estimating the impact of additional stunning-related costs on farm-level profitability. The primary feasibility metric was whether the segment remained profitable after the inclusion of new expenses. Profitability was defined using net margin, calculated as:

Net Margin (%) = (Net Profit ÷ Total Revenue) × 100

Baseline net profit values were obtained from economic performance data in the EU Data Collection Framework (2018–2020), which is reported biannually through STECF [46]. Net profit under stunning scenarios was calculated by subtracting the estimated additional costs of stunning technologies from baseline net profit:

Net Profit (with stunning) = Net Profit (baseline) − Additional Stunning Costs

The revised net margin was then recalculated as:

Net Margin (with stunning) = (Net Profit with stunning ÷ Total Revenue) × 100

Segments were categorized according to economic feasibility thresholds:

- Net Margin (with stunning) > 5%: Economically robust;
- 0–5%: Slim margins;
- <0%: Economically unfeasible.

To test robustness, alternative assumptions about cost absorption and pass-through to consumers were also modeled. The effective stunning cost under partial pass-through was defined as:

Adjusted Stunning Cost = Stunning Cost × (1 − α)

where α is the proportion of the cost passed to consumers (0 ≤ α ≤ 1).

Three scenarios were examined: full absorption (α = 0), full pass-through (α = 1), and partial pass-through (0 < α < 1).

Finally, the robustness of the economic feasibility outcomes was evaluated via sensitivity analyses (see Section 2.7), testing the effect of variations in key parameters.

## 3. Results

### 3.1. Do Stunning Methods Increase Production Costs?

#### 3.1.1. The Total Additional Costs per Enterprise

**Figure 3:**
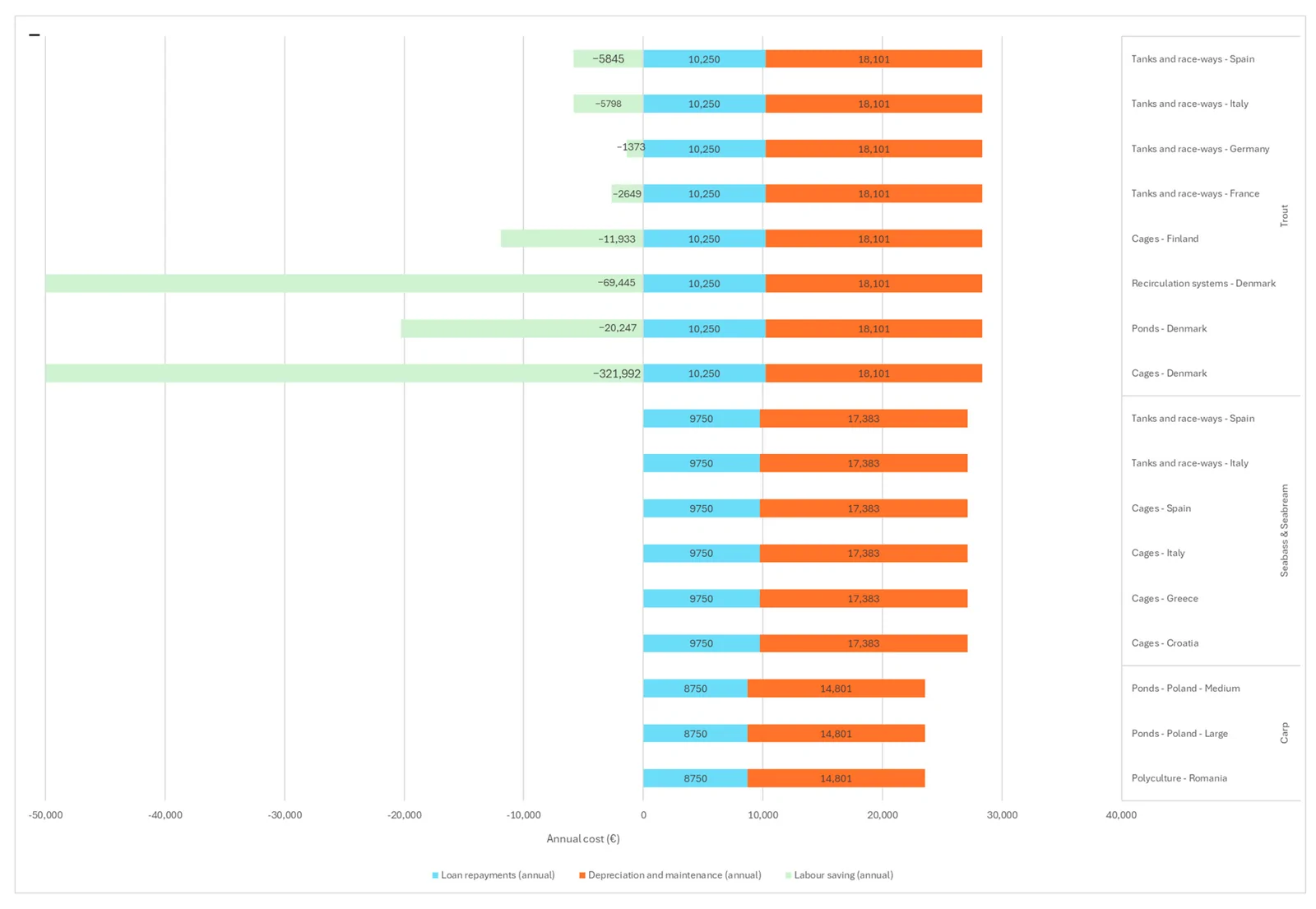
Breakdown of the annual projected costs per enterprise associated with introducing in-water electrical stunning for the 17 segments analysed. For RAS and cage systems in Denmark, the total savings exceed the scale of the figure, so data labels provide the actual figure. There are labor savings for eight out of the 17 segments, and no labor costs for the remaining nine segments. Two country–system–production system-stunning method combinations, Denmark RAS and cages, show an overall saving from introducing stunning mechanisms. To account for inflation, all cost estimates are adjusted to 2024 values in accordance with data from Eurostat on the Harmonized Index of Consumer Prices for the respective years [50].

**Figure 4:**
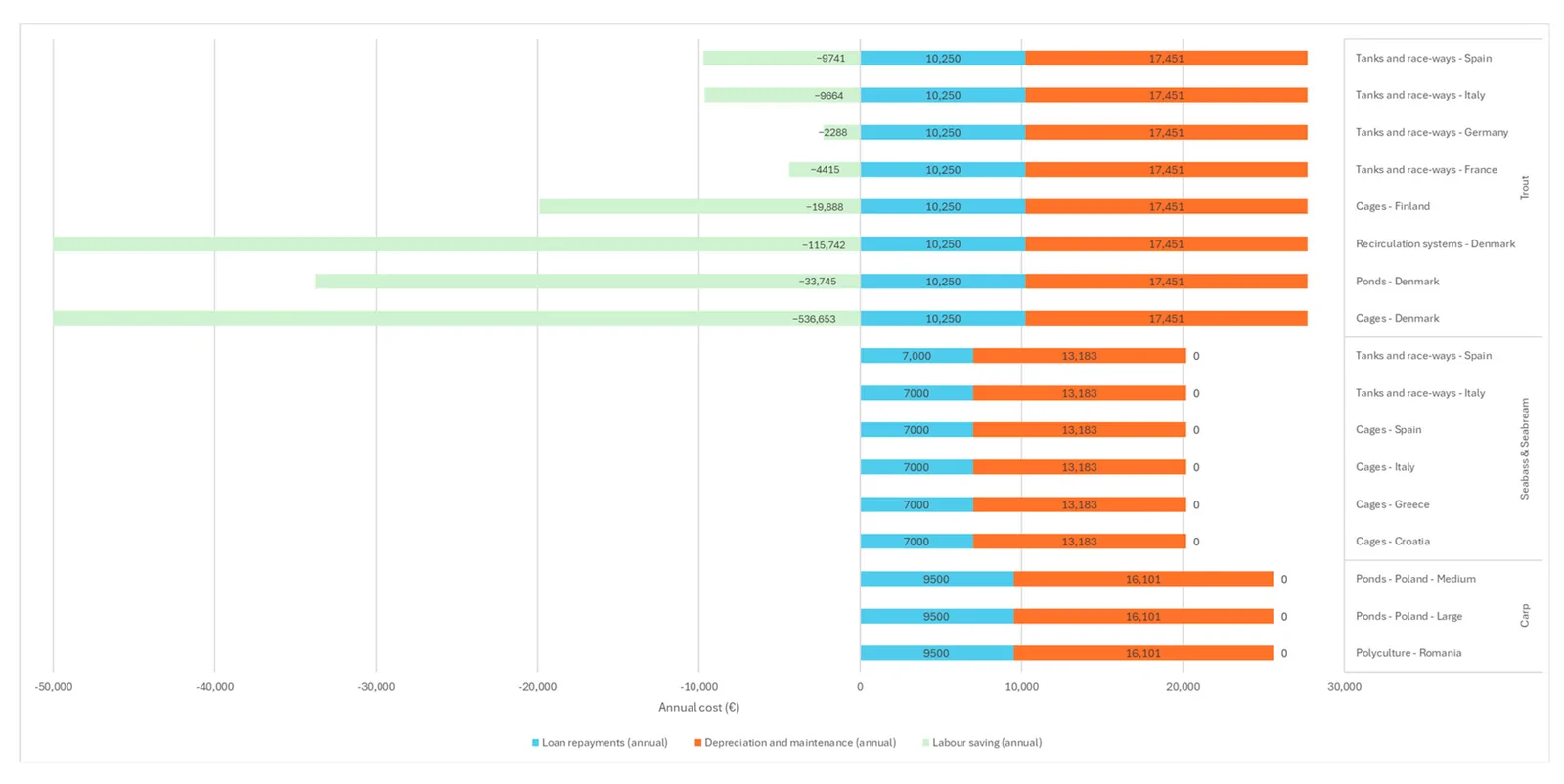
Breakdown of the annual projected costs per enterprise associated with introducing dry electrical stunning for the 17 segments analyzed. For trout RAS and cages in Denmark, the total savings exceed the scale of the figure, so data labels provide the actual figure. There are labor savings for eight out of the 17 segments, and no labor costs for the remaining nine segments. Three country–system–production system-stunning method combinations, Denmark RAS, ponds, and cages, show an overall saving from introducing stunning mechanisms. To account for inflation, all cost estimates are adjusted to 2024 values in accordance with data from Eurostat on the Harmonized Index of Consumer Prices for the respective years [50]. The interquartile ranges for dry electrical stunning (EUR 17,960–EUR 23,286; median EUR 20,183; IQR EUR 5326) and in-water stunning (EUR 22,506–EUR 27,133; median EUR 23,551; IQR EUR 4627) are broadly similar, although the distribution for dry stunning skews slightly lower. This result reflects a modest tendency for dry stunning to include more lower-cost implementations, though overall variation in typical annual costs is comparable between the two methods. Mean values by species and stunning method are provided in Table 6.

Across segments, annual cost changes associated with introducing stunning vary substantially, largely due to differences in labor savings (Figure 3 and Figure 4). Across all segments, additional annual costs from introducing stunning methods range from significant savings (–EUR 508,953) to moderate additional costs (+EUR 27,133). While a few outliers widen the distribution, most values clustered between EUR 20,183 and EUR 27,000 (median EUR 23,551).

**Table 6:** The average (mean) costs per enterprise for dry electrical or in-water electrical stunning, and the overall average.

	Dry Electrical Stunning (EUR)	In-Water Electrical Stunning (EUR)	Overall Average Cost (EUR)
All species	18,390	1234	9812
Carp	25,601	23,551	24,576
Trout	−63,816	−26,559	−45,188
Seabass and Seabream	20,183	27,133	23,658

On average, costs are highest for carp, closely followed by seabass and seabream. Notably, in-water electrical stunning options also tend to be more costly to implement than dry electrical stunning, other than for carp, which is due to the nature of the industry. For example, whilst the carp sector is primarily formed of small producers (see Appendix A), with less resilience to cost increases, the in-water stunning equipment for carp is based at the abattoir and incurs relatively low maintenance and depreciation costs, compared with the in-water stunning for seabass and seabream, which is situated on the harvest boat and is more costly to maintain (see Figure 3).

#### 3.1.2. Cost per Unit of Production

**Figure 5:**
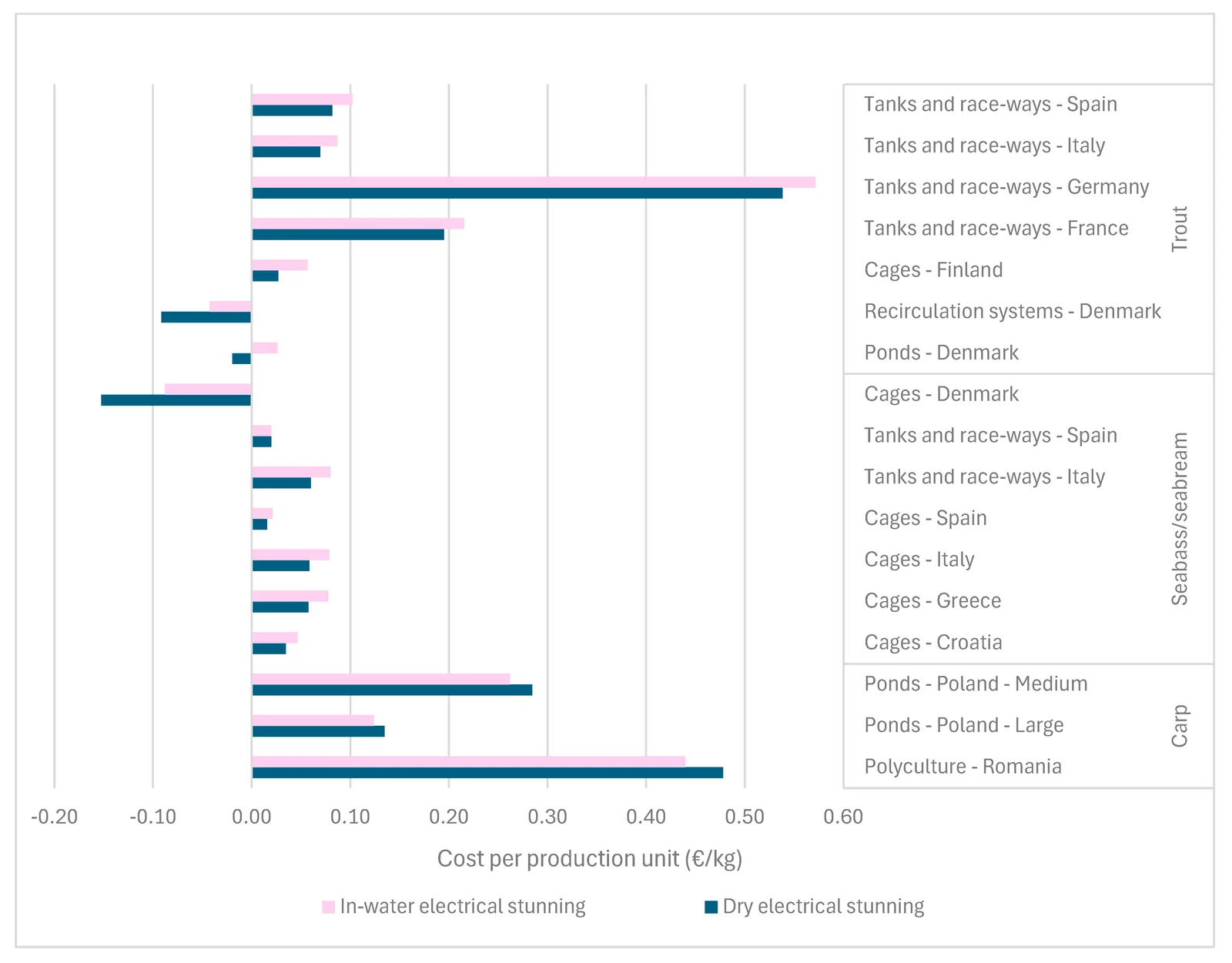
The cost per production unit (EUR/kg) of introducing stunning at slaughter using the ‘Business As Usual’ (BAU) data from STECF [46] as the baseline. To account for inflation, all cost estimates are adjusted to 2024 values in accordance with data from Eurostat on the Harmonized Index of Consumer Prices for the respective years [50].

As with the total additional costs, five country–system–production system-stunning method combinations benefit from a reduction in costs (Figure 5). The majority of segments show moderate increases, with a median of EUR 0.06/kg and an interquartile range (IQR) of EUR 0.03–EUR 0.22 (Figure 5). For dry electrical stunning, the median cost is EUR 0.06/kg (IQR: EUR 0.14), with changes ranging from −EUR 0.15 to +EUR 0.54/kg. In-water stunning shows a similar pattern, with a slightly higher median of EUR 0.08/kg and a wider IQR of EUR 0.17, ranging from −EUR 0.09 to +EUR 0.57/kg. This suggests that while some segments show notable cost reductions or increases, most changes cluster around small-to-moderate positive values. Mean costs by species and stunning method are presented in Table 7.

On average, carp segments once again face the highest cost per kg due to lower production volumes per enterprise. Trout segments have both the lowest (–EUR 0.15/kg) and highest (+EUR 0.58/kg) values, reflecting the diversity of system types within that sector (Figure 5). There is no notable difference between the type of stunning equipment used, and the difference in average cost is minimal (EUR 0.01/kg).

#### 3.1.3. Costs as a Percentage of Current Production Costs

**Figure 6:**
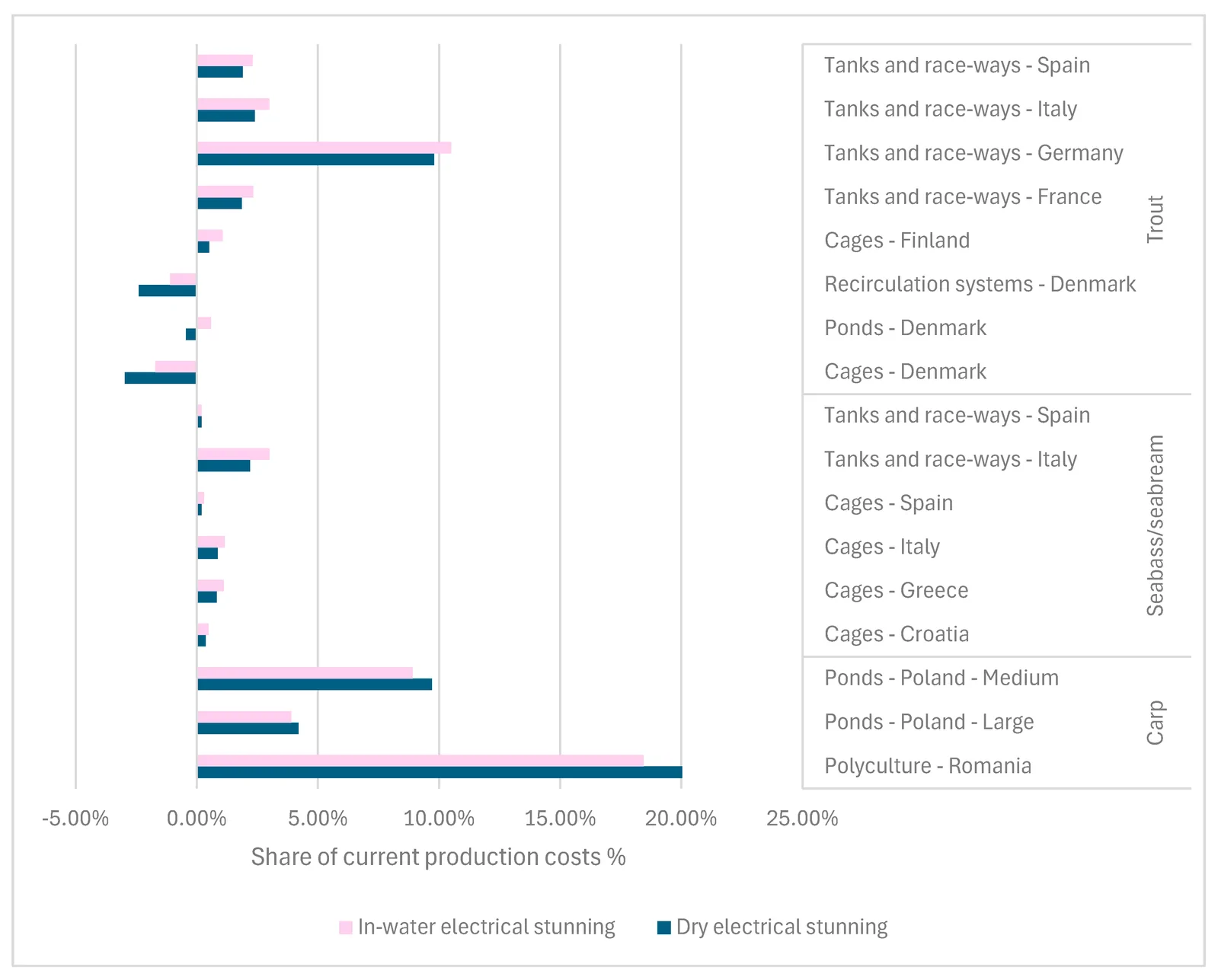
The share of current production costs (%) when introducing stunning at slaughter based on ‘Business As Usual’ (BAU) data from STECF [46] as the baseline. To account for inflation, all cost estimates are adjusted to 2024 values in accordance with data from Eurostat on the Harmonized Index of Consumer Prices for the respective years [50].

When expressed as a percentage of current production costs, stunning-related cost changes range from −2.97% to +20.04% across the segment–method combinations (Figure 6). Median increases are modest, at 1.13% (IQR 2.68%), with most falling between 0.2% and 4.2%. This suggests that while a few categories show large positive or negative changes, most cost shares are concentrated around small-to-moderate proportions of total production costs.

For dry electrical stunning, cost shares range from −2.97% to +20.04%, with a median of 0.86% and an IQR of 2.2%. Comparatively, in-water stunning has a slightly higher median of 1.16% and an IQR of 2.52%, with changes ranging from −1.71% to +18.44%.

As in other cost metrics, the carp sector shows the highest relative cost burden, reflecting the small scale and low margins typical of that industry (see Appendix A). Trout segments again show substantial diversity, with several realizing cost reductions. Table 8 presents the mean values per species and method.

#### 3.1.4. Costs as a Share of the Final Product Price

**Figure 7:**
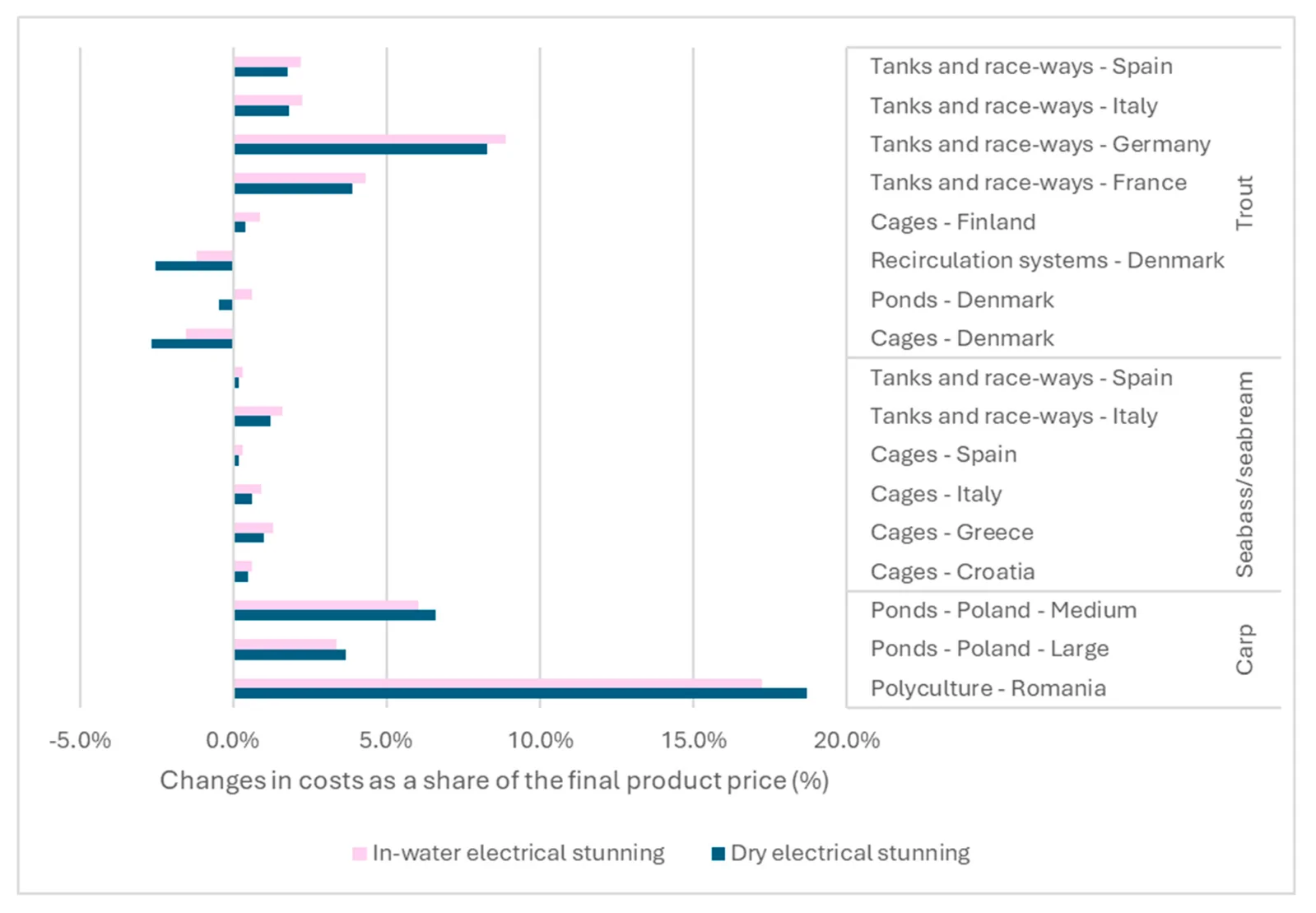
The changes in costs as a share of the final product price (%) when introducing stunning at slaughter based on ‘Business As Usual’ (BAU) data from STECF [46] as the baseline. To account for inflation, all cost estimates are adjusted to 2024 values in accordance with data from Eurostat on the Harmonized Index of Consumer Prices for the respective years [50].

Stunning related costs, as a share of final product price, range from −2.65% (cost savings) to +18.72% across segments. Most increases are small, with a median of 1.6% and an IQR of 3.53% (Figure 7). For in-water stunning, changes range from −1.53% to +17.22% (median: 1.9%; IQR: 3.08%). For dry stunning, values range from −2.6% to +18.72% (median: 1.5%; IQR: 3.53%). These results suggest little difference between the two methods in terms of typical price impact. As with other metrics, carp segments bear the highest relative increases (up to 9.3%), whereas several trout systems show reductions, implying potential for price-neutral or profit-enhancing outcomes. Full species-level breakdowns are shown in Table 9.

#### 3.1.5. Summary of Stunning Cost Impacts

Across the 34 country–species–system-stunning method combinations analyzed (17 segments), introducing stunning results in highly variable cost impacts, depending on the species, production system, and method used. While five combinations show net cost savings from reductions in labor costs, most segments experience moderate increases.

Total additional costs per enterprise typically fall between EUR 20,000 and EUR 27,000, with costs per unit of production clustering around EUR 0.06–EUR 0.08/kg. When expressed relative to existing production costs or final product prices, most segments see additional costs equivalent to less than 5%, with medians of 2.2% and 1.6%, respectively.

The carp sector consistently exhibited the highest relative cost burdens across all metrics, primarily due to small enterprise size and low baseline profitability (see Appendix A). In contrast, trout systems are the most diverse, including both the lowest and highest cost changes across multiple measures. Overall, while the cost of implementing stunning is non-negligible, it remains a small share of the production and sale price for most systems.

### 3.2. How Does Cost Pass-Through Affect Economic Feasibility?

#### 3.2.1. Market-Based Cost Pass-Through

**Figure 8:**
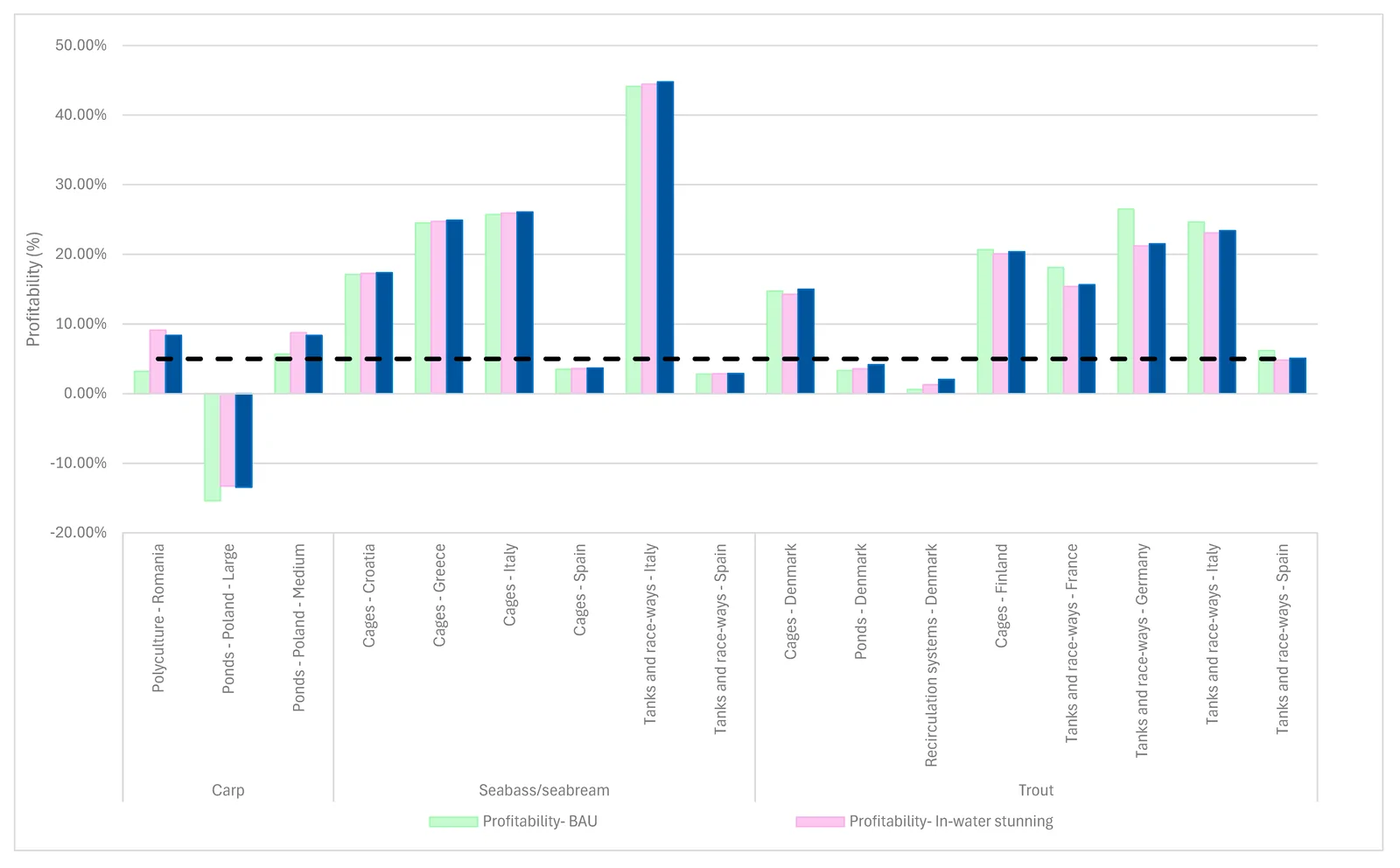
Comparison of profitability before and after implementation of in-water and dry electrical stunning mechanisms for each of the 17 country–species–system segments. Results are shown using cost pass-through based on trade data and market power (i.e., 43% for carp, 40% for trout, and 35% for seabream and seabass). For each segment, the line markers indicate profitability as a percentage of the sale price. The dashed line at 5% represents a minimum robust profit margin threshold.

In the BAU scenario, the finfish aquaculture sector averages EUR 472,419 (mean) profits per enterprise annually, and profitability averages 12.35% (mean). All but one (large carp ponds in Poland) of the 17 segments are profitable (Figure 8). Five segments have slim profit margins of less than 5%, and ten have robust profit margins of above 5%.

Under the market-based cost pass-through scenario (43% for carp, 40% for trout, 35% for seabream and seabass), all but one segment (large carp ponds in Poland) remains profitable following the introduction of either in-water or dry electrical stunning (Figure 8). Segments that were robustly profitable before stunning remain so, while those with slim margins (<5%) see minor changes but remain feasible. The averages, both mean and median, for the profits and profitability are shown in Table 10 for a full and fair comparison of the changes incurred in profits when implementing electrical stunning.

When dry electrical stunning is introduced, there is an increase in the mean annual profit per enterprise of EUR 8257. This is largely due to three of the trout segments (Cages, Ponds, and RAS in Denmark) showing substantial gains (EUR 3657, EUR 53,265, and EUR 307,916). The median profit changes are, however, negative for both stunning types (EUR−13,644 and EUR−10,913), reflecting the influence of the few outliers.

Segment-level results show considerable variation in profit change under both stunning methods. For in-water stunning, changes range from a loss of EUR 47,328 to a gain of EUR 177,653, and for dry stunning, from a loss of EUR 48,496 to a gain of EUR 307,916. However, for both, the ranges are affected by several outliers. Most notably, the Polish carp segment, which was already unprofitable at BAU, incurs the greatest loss once stunning is implemented. Conversely, several trout segments gain from implementing stunning due to labor savings (see Figure 8).

Changes to profitability (%) follow a similar pattern across the segments. For in-water stunning, profitability changes range from a decrease of 9.19% to an increase of 0.98%, while for dry stunning, the range is −9.94% to +1.71%. The majority of declines in profitability are modest: seven segments experience reductions of less than 1% under both methods; four segments see declines between 1 and 5%; two segments (in-water) and one segment (dry) experience a reduction of between 5 and 10%. A small number of segments show improved profitability (two under in-water, three under dry stunning) as a result of operational cost reductions.

#### 3.2.2. Sensitivity Analyses on Cost Pass-Through

Sensitivity analyses were conducted to assess the impact of high- and low-cost pass-through assumptions (0% and 100%) on profits and profitability (Table 11).

Under a 0% cost pass-through scenario with in-water electrical stunning, the segment that was unprofitable (Large Carp Ponds in Poland) at BAU becomes more unprofitable. Eleven segments remain at or above the robust profitability threshold of 5%, one drops slightly below (4.81%), and four maintain slim profit margins (0–5%).

With dry electrical stunning and 0% cost pass-through, the same segment becomes slightly more unprofitable. All other segments remain profitable: 10 exceed the 5% threshold, while 6 fall within the slim margin range (see Table 11).

Under a 100% cost pass-through scenario, outcomes closely resemble the market-based scenario, with only one unprofitable segment (Table 11). The distribution of robust and slim-margin segments also remains unchanged from that of BAU. Differences between stunning types are minimal, typically less than 1% in profit margin terms.

Notably, in two of the trout segments (cages and RAS in Denmark), profitability is marginally higher under the 0% cost pass-through scenario for both stunning types, compared to the market-based pass-through rate of 40%. This reflects minor shifts in the relationship between profit growth and sales price inflation rather than substantive differences in economic outcomes.

#### 3.2.3. Sensitivity Analyses on Increased Annual Costs

To determine the impact of higher-than-expected annual operating costs from implementing stunning, such as increases in energy use, maintenance costs, or depreciation, profitability was re-evaluated under scenarios where total additional annual costs were increased by 10% and 20% relative to the base stunning scenario (Table 12).

Eleven segments continue to remain above the robust profitability threshold of 5%, even with an additional 10 or 20% in annual costs (highlighted green in Table 12). One segment is just below the robust profitability threshold, another four maintain slim profit margins (0–5%), and the same segment showing a loss at BAU shows an even greater loss with the increased annual costs.

Whilst profitability declines progressively with increased annual cost, the impact is moderate for most segments (i.e., 1–3% drop from BAU to +20% scenario). From BAU to +10%, declines range from negligible to ~10%, with the largest drops occurring in the carp segments. From BAU to +20%, the total drop in profitability ranges from under 1% in resilient segments (e.g., seabass/seabream systems) to over 11% in more vulnerable ones (e.g., carp polyculture in Romania). Overall, cost increases have a measurable but variable impact, with well-performing systems showing strong resilience and maintaining solid profitability even under higher cost pressures.

#### 3.2.4. Summary of Cost Pass-Through Impacts

Overall, the economic feasibility of introducing stunning was minimally influenced by cost pass-through assumptions (Table 11). Under market-based pass-through rates (35–43%), all but one segment remains profitable, with most retaining their pre-stunning profitability levels. When no costs are passed on (0% scenario), profitability declines in most segments but remains positive in all but one. Full pass-through (100%) has effects similar to the market-based scenario.

Overall, 11 of the 17 segments maintain robust profitability (>5%) under all scenarios, and 16 out of 17 segments remain profitable. This indicates that stunning is likely to be economically feasible across the majority of the EU aquaculture sector, even in the absence of full cost recovery. Furthermore, whilst profitability generally declined as costs increased in the +10/20% additional annual costs scenarios, most segments only experienced modest reductions of 0.2 to 1% between each step, with resilient segments maintaining stable margins (Table 12).

These results provide a detailed assessment of the cost implications and economic feasibility of implementing stunning across 17 country–species–system segments. Together, they offer a robust evidence base to inform policy decisions, highlighting the financial impact under varied cost structures and market conditions. The following section discusses these findings in the context of existing research, potential limitations, and broader implications for animal welfare policy in EU aquaculture.

## 4. Discussion

This study aimed to assess the economic feasibility of implementing stunning at slaughter for four of the main finfish species in EU aquaculture. It is important to note that electrical stunning may not fully eliminate welfare concerns for all individuals [33,34,35,36,37], but it is currently among the most feasible interventions available [32].

The aim of the study was achieved by analyzing (1) whether stunning at slaughter increases production costs, (2) how the degree of cost pass-through affects economic feasibility, and (3) whether any increased costs can be absorbed.

Implementing electrical stunning in aquaculture generally results in increased costs, although these are modest for most systems. In fact, profitability remains robust across the majority of segments, even under the highest-cost assumptions, including a 20% increase in additional costs and a zero-cost pass-through scenario. Some segments even benefit from net cost savings due to reduced labor requirements. Overall, the carp sector shows the greatest vulnerability, due mostly to its current precarious economic status, and the trout sector shows the highest variability, with several segments seeking to gain considerably from introducing stunning.

### 4.1. Comparison with Previous Studies

The results expand on prior economic assessments of stunning in European aquaculture by applying more recent and granular economic data, updated cost assumptions, and segment-level profitability modeling, including robust sensitivity analyses across multiple cost scenarios.

Overall, this study confirms the general pattern seen in the EC and EA/AA studies, in that the cost of implementing stunning for finfish in EU aquaculture is modest for most species and systems (Table 13). However, the more granular structure used in this study reveals greater variation in the outcomes, particularly for trout and carp. These differences reflect the production system type used, as well as the cost structure, scale, and baseline profitability across the 17 segments analyzed.

The wider cost impacts seen for carp in the EC study are likely driven by assumptions around farm size and system type, whereas the narrower ranges in the current study reflect the system-level modeling used. For both studies, carp is the highest-cost sector due to its low production scale and limited price margins (Appendix A). In all three studies, the cost impacts for seabass and seabream tend to be the lowest, which is consistent with the sector’s relatively high production volumes, meaning better price points (Appendix A). Trout shows the most variation across all three studies, with the EC and EA/AA studies reporting modest increases and the current study finding both the highest and the lowest costs for this species. This finding reflects the high diversity in trout systems (e.g., RAS versus cages) and their associated input costs and labor needs.

In the previous studies, wage levels were assumed at a specific level, whereas in the current study, the actual wages reported by segment in the STECF data were used to ensure a more granular approach. Under both wage level methodologies, several fleet segments show net cost reductions from electrical stunning as a result of labor savings. The extent of labor savings depends on the nature of the production segment. Systems with high manual input, for example, small tanks or raceways requiring individual fish handling, or those countries with higher labor costs, benefit more from stunning, as it reduces handling time per batch and therefore labor costs.

### 4.2. Evaluation of Modeling Assumptions and Real-World Feasibility

A number of methodological assumptions were made in this study (Table 5). Such assumptions are a necessary component of analysis and are similar to those used in previous research [2,40,41]. Firstly, the model assumed that a proportion of the additional costs would be passed along the supply chain, in line with trade-based estimates of market structure. These were tested using 0% and 100% cost pass-through sensitivity analyses. Whilst re-world cost recovery will vary across segments, a 0% cost pass-through is unlikely to occur for this sector, as, unlike with salmon, the remaining EU finfish consumption is predominantly supplied by EU production (see Section 1). This strengthens the assumption that some costs will be passed to the consumer, as assumed in the EC and EA/AA studies.

The second assumption was that the average enterprise is characteristic of the industry. Whilst such an assumption is standard practice for widespread analyses, aligns with previous studies, and is the only option due to data being reported as a total for each segment [2,40,41], it is important to note the potential implications. Aquaculture enterprises can vary widely in scale and structure, particularly in the carp farming sector, which is comprised of small-scale and part-time enterprises [48]. In such sectors, using an “average” can mask the variability in resilience or vulnerability. However, for most intensive aquaculture systems, including trout in RAS and seabass in cages, the average enterprise values seen in the STECF dataset appear to closely reflect the industry [2]. For these segments, the assumption is, therefore, more robust, offering a realistic picture of profitability and cost structures under stunning protocols.

The model also assumed that the enterprises had not already implemented stunning equipment and practices. This assumption was supported by sectoral reports indicating that stunning is not widely used for carp, trout, seabass, and seabream in the EU [2]. Conversely, stunning is more routine for salmon, so the species was excluded from the study [2,7,45]. Therefore, whilst it is possible that a small number of enterprises may have adopted stunning, the overall prevalence remains low, meaning the assumption reflects the current state of the industry for these species.

The fourth assumption was that enterprises purchase stunning equipment individually. However, this assumption is highly unlikely because the equipment would sit idle most of the time. In reality, it is likely that stunners would either be shared between enterprises, be installed only in specific processing plants (used by multiple enterprises), or be hired or sourced on demand by a third party. In these alternative scenarios, the costs incurred would be considerably lower than what is modeled, as the capital costs and maintenance and depreciation burdens per enterprise would be reduced. However, this conservative approach was deliberate in order to determine economic feasibility in a worst-case scenario, thereby providing a robust test of economic feasibility. Consequently, real-world implementation of stunning equipment under more flexible or cooperative arrangements would likely be even more cost-effective.

In relation to this, it was also assumed that equipment would be financed at a 5% interest rate rather than purchased outright or with a low-cost financing option. This assumption reflects a reasonable, market-based estimate of commercial lending rates and aligns with previous economic modeling in aquaculture contexts [2,40,41]. In reality, larger enterprises may be able to purchase the equipment outright, which would avoid interest rates, resulting in a lower financial impact. Others may access low-interest financing through schemes such as the European Maritime Fisheries and Aquaculture Fund (EMFAF), which provides low-cost financing for improvements to aquaculture enterprises. And some may face higher borrowing costs due to limited credit access. Therefore, the 5% financing assumption provides a middle-ground estimate that reflects common practice whilst not overstating feasibility.

The sixth assumption was that product quality and, therefore, product price would remain unchanged. While electrical stunning can lead to carcass damage, this can be avoided or minimized to avoid any detriment to product quality [2]. Furthermore, fish experience less stress at slaughter when stunned, which can positively impact product quality [27,43]. In addition, there may be market opportunities from introducing higher welfare issues, especially as consumers value higher welfare initiatives [53]. Consequently, this assumption provides a cautious baseline for assessing economic feasibility, as improvements in quality or marketability are not accounted for.

Lastly, the seventh assumption was that changes to energy costs would be insignificant. This assumption is supported by previous studies, where manufacturers did not report energy consumption as a significant cost factor, constituting an average of 3.3% of operating costs [2,40,41] and where energy impacts were considered marginal in broader assessments of aquaculture operational expenses [39,51,52]. However, sensitivity analyses introducing a 10% and 20% increase in annual operating costs were used to account for any potential variability.

Overall, these seven assumptions are based not only on previous studies and available data but were chosen to reflect realistic, conservative estimates of economic feasibility rather than overstated possibilities. Consequently, the real-world implementation is likely to be more favorable than what is modeled here. In short, these findings provide a solid foundation for policy decisions regarding implementing stunning in finfish aquaculture, which would provide considerable welfare improvements to the hundreds of millions of fish slaughtered annually in the EU.

### 4.3. Implications for Policy and Practice

The findings from this study have clear implications for ongoing policy discussions around the revision of EU animal welfare legislation. The recognition of the need to improve welfare practices for fish has seen considerable uptake globally in recent years [2,4,5,53,54], and the call for species-specific slaughter provisions for farmed fish in the European Commission’s Inception Impact Assessment is an important example of this [46]. Therefore, whilst recognizing the limitations of electrical stunning methods, the current study provides timely and granular evidence to support the implementation of such regulations, as introducing stunning is economically feasible in the vast majority of the EU’s finfish aquaculture sector. In particular, even under the highest cost assumptions, most of the country–species–system segments remained profitable. These results demonstrate that regulatory reform that mandates stunning in this sector can be implemented with limited economic disruption. Continued research and development are needed to ensure that this area continues to strive to find more effective solutions for stunning in aquaculture [55], and so these insights provide a foundation for shaping policy measures that not only mandate stunning but also promote continuous improvements in welfare practices across the sector.

Several policy instruments could support the adoption of stunning in aquaculture systems, especially for the more vulnerable segments. For instance, financial incentives such as capital subsidies, low-interest loans, or tax credits for stunning equipment purchases and maintenance could be implemented, and mobile or centralized stunning units could allow multiple enterprises to effectively share equipment, thereby improving cost efficiency and reducing financial barriers. Such initiatives would not only reduce the economic burden of mandating stunning on lower-margin producers, but they would also help to accelerate and ensure widespread compliance with the new legislation.

### 4.4. Limitations

Whilst the model included a higher level of granularity than previous studies, the economic performance data still used averages based on the country–species–system level, which may not fully account for variation within the sector. Such a limitation is generally unavoidable in light of the available data and the need for generalizability and policy relevance. Another limitation is that the model did not account for the dynamism of the market, including changes in consumer demand, price elasticity, and supply chain behaviors in light of increased production costs or improved welfare claims. To minimize this impact, cost pass-through was tested under multiple scenarios. However, pricing outcomes may differ in reality due to changing market conditions and consumer sentiments. Lastly, as discussed in Section 1, there are potentially indirect benefits that are not considered, such as improved product quality and reputational gains, which could potentially offset some of the additional costs in the long term. As such, despite these limitations and assumptions, the findings still offer a conservative estimate of economic feasibility for EU aquaculture, providing a robust baseline for informing policy revision.

Additionally, while stunning reduces stress at slaughter, it does not guarantee complete welfare improvement for all individuals or species, and effectiveness depends on correct application, maintenance of equipment, and operator training [7,32,37,38]. Electrical stunning has known issues with maintenance of unconsciousness, and as such, some individuals may regain consciousness before death, introducing considerable welfare concerns [33,34,35,36,37]. Further research is urgently needed to find suitable methods that are reliable and consistent across species, systems, and individuals [55]. However, the findings from this assessment showcase how introducing stunning can be economically feasible, removing a perceived barrier to its implementation.

### 4.5. Future Research

While this study offers robust evidence of economic feasibility, tested through several sensitivity analyses, further research can support the uptake and implementation of stunning in EU aquaculture. For instance, studies tracking the actual costs, benefits, and adoption rates of implementing stunning in various production settings, such as in salmon production, would help validate the model’s assumptions and promote broader uptake outside the EU. Insights into producer perceptions, barriers to uptake, and communication strategies would also help support the roll-out of new welfare regulations across the EU aquaculture sector.

In addition, to provide greater clarity on the potential for implementation, similar assessments should be undertaken regarding other prospective methods when suitable data become available. More broadly, whilst we have chosen to determine the economic feasibility of introducing electrical stunning, there is a clear need for the development and commercial testing of stunning methods, including the use of percussive methods on a wider range of species, to address the issues associated with electrical stunning and to improve welfare outcomes for farmed fish.

## 5. Conclusions

To date, this study provides the most detailed and comprehensive economic analysis of implementing electrical stunning for farmed finfish in the EU. By using updated cost data and granular modeling across 17 country–species–system segments, the results show that while stunning does result in additional costs, these are generally modest and can be absorbed or passed through without significantly impacting profitability. Even under the highest cost assumptions, including full cost absorption, no price premiums, individual equipment ownership, and no financial support, the majority of segments remain economically robust, with some even experiencing cost savings through improved labor efficiency.

These findings offer timely, evidence-based support for developing species-specific slaughter provisions under the ongoing EU animal welfare legislation revision. While current electrical stunning methods are not ideal in terms of welfare outcomes, this assessment demonstrates that, contrary to common perceptions, introducing stunning is economically feasible. This removes a major barrier to implementing effective stunning systems and supports continued research and development on improving stunning systems. Overall, the results show that stunning is economically feasible in most aquaculture contexts, aligning ethical progress with commercial feasibility.

## Figures and Tables

**Table 1:** Key methodological differences between the EC [28], EA/AA [40,41], and the current study.

Aspect	European Commission (EC) Study [28]	Essere Animali and Animals Ask [40,41]	Current Study and Rationale
Data set year(s)	2013 only	2016–2018	2018–2020
Years of data used	One year	Three years	Three years used to improve representativeness and reliability
Granularity of production data	Country–species	Largest segment for each country and species	Country–species–system. All countries and species above EUR 20 million in annual sales (17 segments) (e.g., trout; tank vs. recirculation)
Wages (used to calculate labor costs)	Estimated averages (EUR 25 k, EUR 50 k, or EUR 75 k/FTE)	Estimated based on the literature	Based on actual wages for each segment
Cost–distribution assessment	Assessed as production costs only	Assumed full pass-through to consumers	Evaluates multiple distribution scenarios
Feasibility assessment	Not assessed	Not assessed	Economic feasibility was assessed to ensure producers maintain profitability

**Table 2:** Research questions and key economic considerations for assessing the feasibility of stunning at slaughter in EU fish aquaculture.

Research Question	Considerations Included in the Study Scope	Considerations Outside of the Scope of This Study
Do stunning methods increase production costs?	Economic costs (financing, depreciation, maintenance	Available subsidies
	Economic savings (labour)	Cost sharing of equipment
2. How does the degree of cost pass-through affect economic feasibility?	Modelling profitability under different cost pass-through scenarios	Price premium for stunning
3. Can any increased costs be absorbed?	Enterprise profitability	
Outcome: Determination of economic feasibility		

**Table 3:** The 17 segments included in the current study, as determined by those country–species–production system segments that were over >EUR 20 million in annual production value. The EC study did not break down the analysis into segments and only used combinations of species and countries. The EA/AA study used the largest segment for each country and species in its analysis. Details are provided in the last column for all relevant segments.

Country	Species	Production System	Covered in Previous Studies and Level of Granularity?
Croatia	Seabass and Seabream	Cages	No
Denmark	Trout	Cages	In the EC study as trout production in Denmark (system not specified).
Denmark	Trout	Ponds	In the EC study as trout production in Denmark (system not specified).
Denmark	Trout	Recirculation systems (RAS)	In the EC study as trout production in Denmark (system not specified).
Finland	Trout	Cages	No
France	Trout	Tanks and raceways	In the EC study as trout production in France (system not specified).
Germany	Trout	Tanks and raceways	No
Greece	Seabass and Seabream	Cages	In the EC study as seabass and seabream production in Greece (system not specified). Included in the EA/AA study as a segment.
Italy	Seabass and Seabream	Cages	In the EC study as seabass and seabream production in Italy (system not specified). Included in the EA/AA study as a segment.
Italy	Seabass and Seabream	Tanks and raceways	In the EC study as seabass and seabream production in Italy (system not specified). Excluded from the EA/AA study, in favor of the cages segment.
Italy	Trout	Tanks and raceways	In the EC study as trout production in Italy (system not specified). Included in the EA/AA study as a segment.
Poland	Carp	Large carp ponds	In the EC study as carp production in Poland (system not specified).
Poland		Medium carp ponds	In the EC study as carp production in Poland (system not specified).
Romania	Carp	Carp polyculture	In the EC study as carp production in Romania (system not specified).
Spain	Seabass and Seabream	Cages	In the EC study as seabass and seabream production in Spain (system not specified).
Spain	Seabass and Seabream	Tanks and raceways	In the EC study as seabass and seabream production in Spain (system not specified).
Spain	Trout	Tanks and raceways	No

**Table 4:** Equipment costs used in the current study, including the purchase cost, depreciation rate, and maintenance rate of each equipment. Negative numbers for labor savings indicate cost savings due to improved efficiency. To account for inflation, all cost estimates have been adjusted to 2024 values in accordance with data from Eurostat on the Harmonized Index of Consumer Prices for the respective years [50].

Equipment	Purchase Cost (EUR)	Annual Loan Repayments (EUR)	Depreciation Rate	Maintenance Rate	Labor Saving (Hours /Tonne)	Source
Pump (2–3 tonnes/h)	30,000		10%	2%	-	EA/AA based on quotes from FAIVRE and VAKI
Pump (20–30 tonnes/h)	55,000		10%	2%	-	EA/AA based on quote from FAIVRE
Dewater and singulation unit before stunning	50,000		10%	2%	-	EC based on equipment manufacturers
Dewater unit after stunning	5000		10%	2%	-	EC based on equipment manufacturers
Electro stunner after dewatering (1–20 tonnes/h)	56,000		5%	4%	−2.5 (for trout)	Response received from Optimar (current study)
Electro stunner in water in an abattoir (5–10 tonnes/h)	70,000		10%	1%	-	EC based on equipment manufacturers
Electro stunner in water in an abattoir (20 tonnes/h)	100,000		10%	1%	−1.5 (for trout)	EA/AA based on quote from Ace Aquatec
Electro stunner in water on harvest boat (5 tonnes/h)	120,000		10%	1%	-	EA/AA based on quote from Ace Aquatec

**Table 5:** Methodological assumptions used in the study, including the rationale for adoption.

Methodological Assumptions	Rationale
Price transmission is assumed to be 0.5* EU production share of the EU market	EU consumption of the four species is primarily supplied by EU production, meaning that EU enterprises will likely pass on some of the increased cost through higher prices. Sensitivity analyses were performed on the cost pass-through.
The average enterprise is characteristic of the industry	Such an assumption is standard practice for widespread analyses, and was also assumed in previous studies [2,40,41].
Enterprises have not implemented stunning equipment and practices	For the species selected, it is recognized that stunning is not widely used [2]. Stunning is, however, more common for Atlantic salmon [2,44,45], and so this species was excluded from the study.
Enterprises purchase and use stunning equipment individually	As there are long periods during which stunning equipment is not needed, it is highly unlikely that enterprises will purchase and use the equipment individually. Instead, it is highly probable that such equipment is shared among enterprises. However, this assumption was made for methodological simplicity, as the impact of this assumption being violated would only increase economic feasibility and lower costs, rather than increase them.
Product quality, and therefore product price, are unchanged	Whilst electrical stunning can lead to carcass damage, according to the European Commission, this can be avoided or minimized with increasing understanding of required stunning specifications [2].
Equipment is financed at a 5% interest rate rather than purchased outright or with a low-cost financing option	The European Maritime, Fisheries and Aquaculture Fund (EMFAF) provides low-cost financing for improvements to aquaculture enterprises, and so enterprises may have access to a zero-interest loan for stunning equipment. This would lower the costs of implementing stunning by approximately one-third (varied across segments). Similarly, purchasing the equipment outright would also remove the additional interest rate costs.
Changes to energy costs are insignificant	Changes to energy costs are insignificant as they were not reported by manufacturers in previous studies [2,40,41], and are reported to be marginal elsewhere [39], especially in comparison with the average energy consumption of aquaculture in general [51,52]. Sensitivity analyses were performed to test the impact of increased costs.

**Table 7:** The average (mean) costs per unit of production (EUR/kg) for dry electrical or in-water electrical stunning, and the overall average.

	Dry Electrical Stunning (EUR)	In-Water Electrical Stunning (EUR)	Overall Average Cost (EUR)
All species	0.11	0.12	0.12
Carp	0.30	0.28	0.29
Trout	0.11	0.15	0.13
Seabass and Seabream	0.01	0.03	0.02

**Table 8:** The average (mean) changes in terms of the share of current costs of production for dry electrical or in-water electrical stunning, and the overall average.

	Dry Electrical Stunning (%)	In-Water Electrical Stunning (%)	Overall Average Cost (EUR)
All species	2.90	3.20	3.05
Carp	11.31	10.41	10.86
Trout	1.33	2.12	1.73
Seabass and Seabream	0.77	1.04	0.91

**Table 9:** The average (mean) changes in terms of the share of the final product for dry electrical or in-water electrical stunning, and the overall average.

	Dry Electrical Stunning (%)	In-Water Electrical Stunning (%)	Overall Average (%)
All species	2.54	2.83	2.69
Carp	9.66	8.88	9.27
Trout	0.61	0.84	0.73
Seabass and Seabream	1.32	2.05	1.69

**Table 10:** Summary statistics for profit and profitability across BAU and stunning scenarios with the cost pass-through based on trade data and market power (i.e., 43% for carp, 40% for trout, and 35% for seabream and seabass).

	BAU	In-Water Electrical Stunning	Change From BAU—In-Water	Dry Electrical Stunning	Change from BAU—Dry
Profit per enterprise (EUR)					
Mean	472,419	468,680	−3739	480,676	8257
SD	661,737	700,782	-	727,095	-
Median	246,005	232,361	−13,644	235,092	−10,913
IQR	716,845	705,006	-	700,069	-
Range	−26,600 to 2,618,856	−73,928 to 2,796,509	-	−75,096 to 2,926,773	-
Profitability (%)					
Mean	15.68	13.35	−2.33	13.53	−2.15
SD	12.60	13.09		13.16	
Median	17.54	14.24	−3.30	14.97	−2.57
IQR	20.73	17.57		17.31	
Range	−4.86 to 45.73	−13.26 to 44.44		−13.54 to 44.77	

**Table 11:** Segment level profitability (%) when implementing in-water or dry electrical stunning under base stunning scenario (market-based cost-pass through; 43% for carp, 40% for trout, and 35% for seabream and seabass, and two sensitivity scenarios of 0% and 100% cost pass-through) and in comparison to BAU (business as usual; no stunning implemented). The segments that show robust profitability (>5%) in all scenarios are shaded orange. Note the marked differences in the values for seabass/seabream tanks and race-ways in Spain versus Italy, which are a result of differences in the sectors themselves (see Appendix A).

		BAU (%)	0% Cost Pass-Through (%)		100% Cost Pass-Through (%)		Market-Based Pass-Through (%)	
			In-Water	Dry	In-Water	Dry	In-Water	Dry
Carp	Polyculture—Romania	18.30	3.20	1.91	15.89	15.71	9.11	8.36
Carp	Ponds—Poland—Large	−4.86	−15.40	−15.74	−10.60	−10.56	−13.26	−13.45
Carp	Ponds—Poland—Medium	17.40	5.70	5.05	12.47	12.40	8.75	8.35
Seabass/seabream	Cages—Croatia	17.54	17.10	17.24	17.47	17.49	17.26	17.33
Seabass/seabream	Cages—Greece	25.33	24.50	24.71	25.12	25.17	24.72	24.88
Seabass/seabream	Cages—Italy	26.54	25.70	25.91	26.32	26.38	25.91	26.07
Seabass/seabream	Cages—Spain	3.80	3.50	3.59	3.79	3.79	3.61	3.66
Seabass/seabream	Tanks and race-ways—Italy	45.73	44.10	44.54	45.01	45.19	44.44	44.77
Seabass/seabream	Tanks and race-ways—Spain	3.00	2.80	2.83	2.99	2.99	2.84	2.88
Trout	Cages—Denmark	13.25	14.70	15.83	13.45	13.61	14.24	14.97
Trout	Ponds—Denmark	3.91	3.30	4.33	3.88	3.92	3.56	4.17
Trout	Recirculation systems—Denmark	0.61	1.70	2.94	0.62	0.62	1.27	2.04
Trout	Cages—Finland	20.63	19.80	20.23	20.46	20.55	20.05	20.35
Trout	Tanks and race-ways—France	18.10	14.00	14.36	17.38	17.44	15.35	15.61
Trout	Tanks and race-ways—Germany	26.48	18.80	19.23	24.59	24.69	21.18	21.48
Trout	Tanks and race-ways—Italy	24.64	22.40	22.83	24.10	24.20	23.07	23.38
Trout	Tanks and race-ways—Spain	6.17	4.00	4.43	6.04	6.07	4.81	5.08

**Table 12:** Segment-level profitability (%) under the base stunning scenario and two sensitivity scenarios with increased annual costs (+10% and +20%), intended to reflect potential rises in operational expenses, such as energy, maintenance, or depreciation. Profitability is calculated as net margin (net profit as a percentage of revenue). BAU = business as usual (no stunning implemented). The segments that show robust profitability (>5%) in all scenarios are shaded green. Note the marked differences in the values for seabass/seabream tanks and race-ways in Spain versus Italy, which are a result of differences in the sectors themselves (see Appendix A).

		BAU (%)	Stunning (Base Scenario at Market-Based Cost Pass-through) (%)		Stunning (+10% Annual Cost) (%)		Stunning (+20% Annual Cost) (%)	
			In-Water	Dry	In-Water	Dry	In-Water	Dry
Carp	Polyculture—Romania	18.30	9.11	8.36	8.25	7.44	7.41	6.53
Carp	Ponds—Poland—Large	−4.86	−13.26	−13.45	−13.48	−13.69	−13.70	−13.92
Carp	Ponds—Poland—Medium	17.40	8.75	8.35	8.29	7.86	7.84	7.38
Seabass/seabream	Cages—Croatia	17.54	17.26	17.33	17.2	17.31	17.20	17.29
Seabass/seabream	Cages—Greece	25.33	24.72	24.88	24.66	24.83	24.60	24.79
Seabass/seabream	Cages—Italy	26.54	25.91	26.07	25.85	26.03	25.79	25.98
Seabass/seabream	Cages—Spain	3.80	3.61	3.66	3.59	3.64	3.57	3.63
Seabass/seabream	Tanks and race-ways—Italy	45.73	44.44	44.77	44.31	44.68	44.18	44.58
Seabass/seabream	Tanks and race-ways—Spain	3.00	2.84	2.88	2.83	2.87	2.81	2.86
Trout	Cages—Denmark	13.25	14.24	14.97	14.34	15.14	14.44	15.31
Trout	Ponds—Denmark	3.91	3.56	4.17	3.52	4.20	3.49	4.22
Trout	Recirculation systems—Denmark	0.61	1.27	2.04	1.34	2.19	1.41	2.33
Trout	Cages—Finland	20.63	20.05	20.35	19.99	20.32	19.93	20.30
Trout	Tanks and race-ways—France	18.10	15.35	15.61	15.08	15.36	14.81	15.12
Trout	Tanks and race-ways—Germany	26.48	21.18	21.48	20.67	21.00	20.16	20.51
Trout	Tanks and race-ways—Italy	24.64	23.07	23.38	22.91	23.26	22.76	23.13
Trout	Tanks and race-ways—Spain	6.17	4.81	5.08	4.68	4.98	4.54	4.87

**Table 13:** The results from this study compared with those from the EC and EA/AA studies.

Species	Measure	EC Study	EA/AA Study	This Study
Carp	Cost per unit (EUR)	0.06 to 0.67	-	0.12 to 0.48
	Share of current price (%)	3 to 28	-	4 to 20
Seabass and Seabream	Cost per unit (EUR)	0.04 to 0.13	0.06 to 0.07	−0.15 to 0.08
	Share of current price (%)	0 to 2	1	0 to 3
Rainbow trout	Cost per unit (EUR)	−0.06 to 0.24	0.06 to 0.07	−0.09 to 0.57
	Share of current price (%)	−2 to 8	2	−3 to 10

## Data Availability

The original contributions presented in this study are included in the article. Further inquiries can be directed to the corresponding author.

## Appendix A

**Table A1:** Additional information on the scale of each of the species aquaculture sectors in the EU.

Segment	Number of Enterprises	Production per Enterprise (BAU) (kg)	Revenue per Enterprise (BAU) (€)	Costs per Enterprise (BAS) (€)	Profit per Enterprise (BAU) (€)	Profitability (BAU) (%)
Carp Polyculture—Romania	272	53,569	156,338	127,748	28,591	18.3%
Carp ponds—Poland—Large	42	190,000	547,200	607,704	−26,600	−4.9%
Carp ponds—Poland—Medium	81	90,000	305,100	264,106	53,100	17.4%
Carp (all segments)	395	75,546	228,404	206,743	27,748	12.1%
Seabass & Seabream Cages—Croatia	23	584,703	6,791,923	5,600,537	1,191,386	17.5%
Seabass & Seabream Cages—Greece	300	349,799	3,291,141	2,457,579	833,562	25.3%
Seabass & Seabream Cages—Italy	23	345,398	3,196,520	2,348,097	848,423	26.5%
Seabass & Seabream Cages—Spain	25	1,283,135	9,660,380	9,293,618	366,762	3.8%
Seabass & Seabream Tanks and race-ways—Italy	21	179,132	1,689,519	916,822	772,697	45.7%
Seabass & Seabream Tanks and race-ways—Spain	4	1,001,831	11,719,845	11,368,532	351,312	3.0%
Seabass and Seabream (all segments)	396	418,215	3,879,762	3,061,918	817,844	21.1%
Trout Cages—Denmark	4	3,339,710	19,757,767	17,138,911	2,618,856	13.3%
Trout Ponds—Denmark	55	309,080	1,429,840	1,373,988	55,851	3.9%
Trout Recirculation systems—Denmark	17	962,905	3,769,300	3,746,336	22,964	0.6%
Trout Cages—Finland	30	289,281	1,932,769	1,534,046	398,724	20.6%
Trout Tanks and race-ways—France	322	119,412	623,869	510,980	112,889	18.1%
Trout Tanks and race-ways—Germany	151	47,198	350,980	258,056	92,923	26.5%
Trout Tanks and race-ways—Italy	143	259,533	998,385	752,380	246,005	24.6%
Trout Tanks and race-ways—Spain	81	220,050	1,031,533	967,863	63,670	6.2%
Trout (all segments)	802	194,089	945,858	800,733	145,125	15.3%
